# Inhibition of PI3K suppresses propagation of drug-tolerant cancer cell subpopulations enriched by 5-fluorouracil

**DOI:** 10.1038/s41598-017-02548-9

**Published:** 2017-05-23

**Authors:** Kaoru Ishida, Chie Ito, Yukimi Ohmori, Kohei Kume, Kei A. Sato, Yuka Koizumi, Akari Konta, Takeshi Iwaya, Mamoru Nukatsuka, Takashi Kobunai, Teiji Takechi, Satoshi S. Nishizuka

**Affiliations:** 1 Molecular Therapeutics Laboratory, Department of Surgery, Iwate Medical University School of Medicine, Morioka, Iwate, 020-8505 Japan; 2 Division of Biomedical Research and Development, Institute of Biomedical Science, Iwate Medical University, Morioka, Iwate, 020-8505 Japan; 3 Translational Research Laboratory, Taiho Pharmaceutical Co., Ltd., Tokushima, Tokushima, 771-0194 Japan; 4 Center for Applied Proteomics and Molecular Medicine, Institute for Advanced Biomedical Research, George Mason University, Manassas, Virginia 20110 United States

## Abstract

Drug-tolerant cancer cell subpopulations are responsible for relapse after chemotherapy. By continuously exposing the gastric cancer cell line MKN45 to 5-FU for >100 passages, we established a 5-fluorouracil (5-FU)-tolerant line, MKN45/5FU. Orthotopic xenografts of MKN45/5FU cells in the stomach of nude mice revealed that these cells had a high potential to metastasize to sites such as the liver. Levels of phosphorylated phosphatidylinositide 3-kinase (PI3K) increased both in 5-FU-tolerant subpopulations according to the 5-FU dose, and in gastric submucosal orthotopic xenografts of MKN45/5FU cells. Sequential administration of 5-FU and a PI3K inhibitor, GDC-0941, targeted the downstream ribosomal S6 kinase phosphorylation to significantly suppress 5-FU-tolerant subpopulations and tumor propagation of orthotopic MKN45/5FU xenografts. These results suggest that administration of 5-FU followed by GDC-0941 may suppress disease relapse after 5-FU-based gastric cancer chemotherapy.

## Introduction

Despite recent therapeutic advancements, relapse is a major issue for gastric cancer treatment. Multidisciplinary therapy has been considered effective, such as the combination of curative surgery and chemotherapy. One good example is the treatment of advanced-stage gastric cancer, which includes gastrectomy, regional lymph node dissection, and 5-fluorouracil (5-FU)-based chemotherapy^1–3^. Although the treatment regimens vary among countries and institutions, 5-FU is the mainstay of therapy, though the relapse rate remains generally high, even after multidisciplinary treatment^4^. Since no visible tumor mass should be present after surgery with curative intent, disease relapse may be attributed to some very small cancer cell populations that survive and develop drug resistance, despite being continuously exposed to anticancer agents. Therefore, effective treatments to suppress 5-FU resistant cancer cell propagation are urgently needed for relapsed gastric cancer.

The following hypothesis has been posited for drug resistance. First, the pre-existing “relatively” drug-resistant clones are selected in heterogenic cell populations^5^. Second, acquired gene mutations may promote drug resistance^6^. Third, cancer cells may also alter intrinsic molecular pathways in response to stresses induced by anticancer drugs^7^. Taken together, previous reports have suggested that cancer relapse after chemotherapy may have multiple mechanisms that presumably depend on drug types or site of origin. As such, identifying resistance mechanisms associated with drugs that are currently and widely used in practice, such as 5-FU, should provide the most practical information for designing strategies to prevent relapse in cancer patients.

The small populations of cancer cells that survive after chemotherapy can be modeled as drug-tolerant subpopulations that are able to form colonies, which we refer to here as drug-tolerant colonies (DTCs)^8^. In sparsely disseminated cell cultures, these DTCs can emerge in the presence of drugs and form colonies of ~1 mm in diameter. Although not all disseminated cells can form colonies, the number of emerging colonies is constant in a drug concentration-dependent manner. These classical observations have already suggested that the majority of drug resistance is a rapidly induced phenotype. Indeed, we obtained DTCs within 2 weeks of drug exposure, during which time cells can undergo roughly 13 or 14 divisions, as is the case for MKN45 cells^8^. In fact, clinical cancer relapse often show up within a few months, which is much faster than the estimation of the time to genetic alterations accumulate^9^. Therefore, the underlying mechanism of drug resistance is likely due to either pre-existing clones with genetic alterations or prompt adaptation to the drug at protein level in the absence of marked genetic changes^10^.

The current study examined the molecular mechanisms for chemotherapeutic resistance after conventional 5-FU-based therapy. We first assessed 5-FU-tolerant human gastric cancer cell lines at genetic and proteomic levels using cancer-related gene sequencing and proteomic profiling of their DTCs^11^. Subsequently, we investigated how cells that acquired 5-FU-tolerance behaved in a gastric microenvironment using orthotopic xenograft (OX) transplanted into the gastric submucosal layer. The findings we describe here may have strategic impact to reduce resistance of cancer cells triggered by widely-used chemotherapies.

## Results and Discussion

### Cell growth of 5-FU-tolerant cancer cell lines

**Figure 1:**
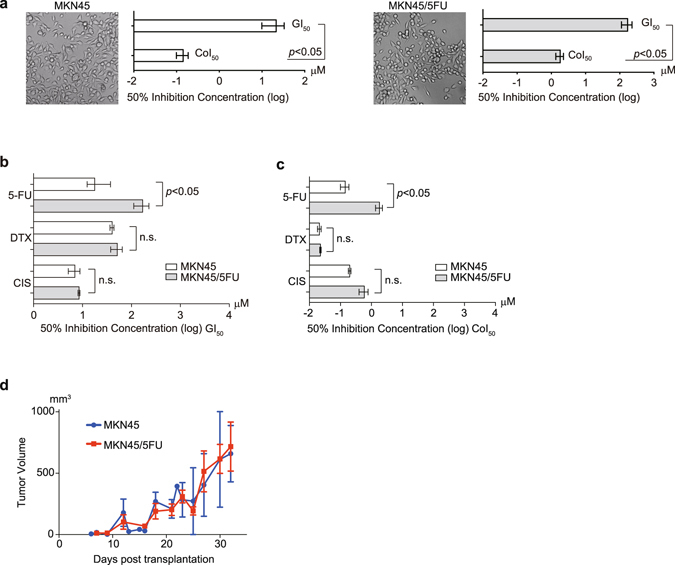
MKN45 and MKN45/5FU cells share similar morphology and growth characteristics. (**a**) Morphology, GI_50_, and CoI_50_ values of MKN45 and MKN45/5FU cell lines. (**b**) GI_50_ values in growth with three different drugs. (**c**) CoI_50_ values in growth with three different drugs. (**d**) MKN45 and MKN45/5FU subcutaneous xenografts in nude mice.

After culturing the parental gastric cancer cell line MKN45 in the presence of continuously escalating concentrations of 5-FU in the culture medium for 1 year, some cells continued to grow despite the presence of the drug^11^. The resulting 5-FU-tolerant cell line MKN45/5FU had similar morphology to MKN45 cells and both cell lines showed a similar trend in 50% inhibition concentration between (GI_50_) and colony formation (CoI_50_) (Fig. 1a). The specific and high tolerance of MKN45/5FU to 5-FU was indicated by the differences in the GI_50_ (Fig. 1b) and CoI_50_ (Fig. 1c) values. Examination of MKN45/5FU treated with cisplatin (CIS) and docetaxel (DTX) did not show cross-resistance to 5-FU (Fig. 1b and c). Subcutaneous transplantation of MKN45 and MKN45/5FU xenografts showed no significant difference in tumorigenicity (Fig. 1d).

### A limited effect of genetic alterations in the acquisition of drug tolerance

Genetic alterations in 191 target regions from 46 cancer-related genes in both MKN45 and MKN45/5FU cells were sequenced using a semiconductor-type next generation sequencer (NGS, Ion PGM, Life Technologies, the accession number for Ion AmpliSeq Cancer Panel used in this study is DRA005227). Of these 46 genes, 7 were altered in both MKN45 and MKN45/5FU cells (Supplementary Table 1). Although target gene mutations often promote drug-resistance^12^, our genotyping seemed to suggest that the drug-tolerant phenotype of MKN45/5FU cells did not result from gene mutations, but instead was due to selection pressure exerted by 5-FU. While “selected” populations could naturally be considered to arise from a pre-existing intrinsically drug-tolerant population^13^, an adaptive response that activates relevant molecular pathways may also allow cancer cells to survive and proliferate in the presence of drugs^7, 10^. In fact, if genetic alterations have a limited effect, then the rapid emergence of recurrent tumors may be easily explained. Relapse after chemotherapy often occurs within a short period of time relative to the chance of occurrence for a gene mutation^5, 14^. Indeed, liver metastasis or peritoneal dissemination can be suppressed temporarily by chemotherapy, but most highly advanced gastric cancer patients experience relapses within a few months, suggesting that drug-resistant populations may have already existed upon chemotherapy initiation and continued to survive during the course of chemotherapy^15^. Taken together, these observations led us to examine the proteomic profile of drug-tolerant cell subpopulations that can be isolated as colonies that emerge in the presence of chemotherapeutic drugs.

### Proteomic characterization of MKN45/5FU cells identifies 5-FU dose-dependent protein expression induction

**Figure 2:**
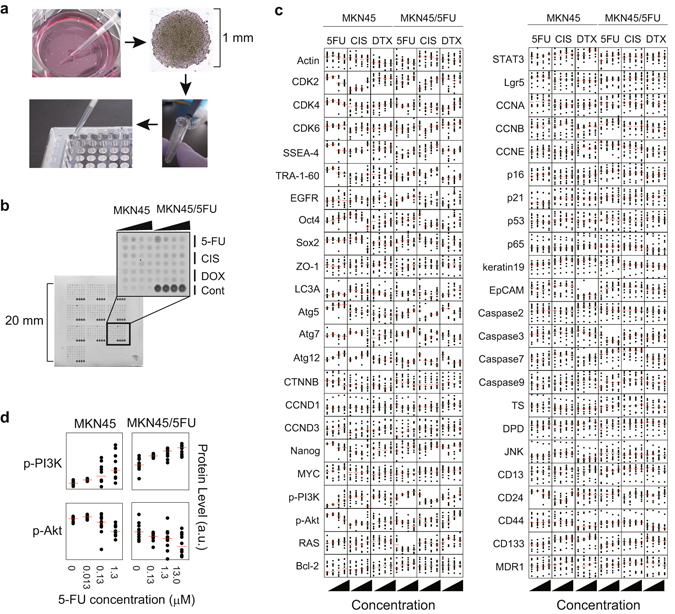
Proteomic characterization of DTCs emerged from MKN45 and MKN45/5FU cells. (**a**) The colony lysate assay involves growing DTCs in the presence of a given drug. Round colonies with a 1 mm diameter were picked individually with a pipette tip and transferred to individual tubes for lysis. Each DTC lysate was then transferred into individual wells of 384-well v-bottom microtiter plates. (**b**) Representative images of CoLA results. Each dot represents a lysate from a single colony that survived in the presence of the indicated drug concentration. Black triangles indicate increasing drug concentration. (**c**) Quantitative monitoring of protein levels in individual DTCs by CoLA. The horizontal axis shows the respective drugs (top) at increasing concentrations (bottom, black triangles). The vertical axis shows the protein level based on the scanned density with an arbitrary unit (a.u.) to maximize the deviation within the vertical axis (i.e., relative protein expression per protein per drug across drug concentration). Red lines for the needle plots indicate mean values of a set of 20 replicated colony lysates. (**d**) Changes in protein levels in individual DTCs for p-PI3K and p-AKT as a function of drug concentration.

In this study, we defined a population of cells that grows adherently in two-dimensional rounded colonies less than 1 mm in diameter after exposure to different drug administration conditions, namely DTCs (Fig. 2a). To quantitatively measure protein expression of individual DTCs, we developed a colony lysate array (CoLA) by selecting individual colonies from cells exposed to a variety of drug conditions based on the reverse-phase protein array (RPPA) technology^16, 17^. Since these 1 mm colonies generally contain only 3,000 to 10,000 cells, comparative techniques, such as fluorescence-activated cell sorting, may not be applicable for quantitative protein measurement of protein species. With CoLA, hundreds to several thousands of individual dots containing lysate from a single colony can be accommodated on a nitrocellulose surface that allows simultaneous and quantitative detection using a set of qualified antibodies (Fig. 2b and Supplementary Table 2).

A cluster analysis of individual DTCs and protein levels revealed two major clusters in the axis of colonies (Supplementary Fig. 1). However, these clusters did not show a significant association with particular drugs or cell types, suggesting that protein expression of DTCs may not be strictly regulated by drugs or cellular origin^8^. In addition, the proteomic profiles of individual DTCs treated with the same drug conditions were highly diverse, which may also imply intrinsic heterogeneity among DTCs.

We next assessed whether levels of 50 proteins in DTCs of MKN45 and MKN45/5FU were associated with drug administration conditions, with particular focus on the drug concentration (Fig. 2c). Among 50 proteins, phosphorylated phosphatidylinositide 3-kinase (p-PI3K) and p-Akt were identified as 5-FU-specific dose-responding proteins in MKN45 and MKN45/5FU cells (Fig. 2d). Of note, CD133 also increased concurrent with 5-FU concentration, which is consistent with the finding by Zhu *et al*. that CD133^+^ gastric cancer cells in a growth suppression assay had greater 5-FU resistance and growth suppression compared to CD133^−^ cells^18^. Notably, a point mutation in *PIK3CA* codon 707 in the region encoding the p110α subunit of PI3K has been found in both MKN45 and MKN45/5FU cells (Supplementary Table 1). This mutation is located in the catalytic domain, which may cause constitutive PI3K activation^19^.

### An OX model of MKN45/5FU reveals acquisition of a malignant phenotype

**Figure 3:**
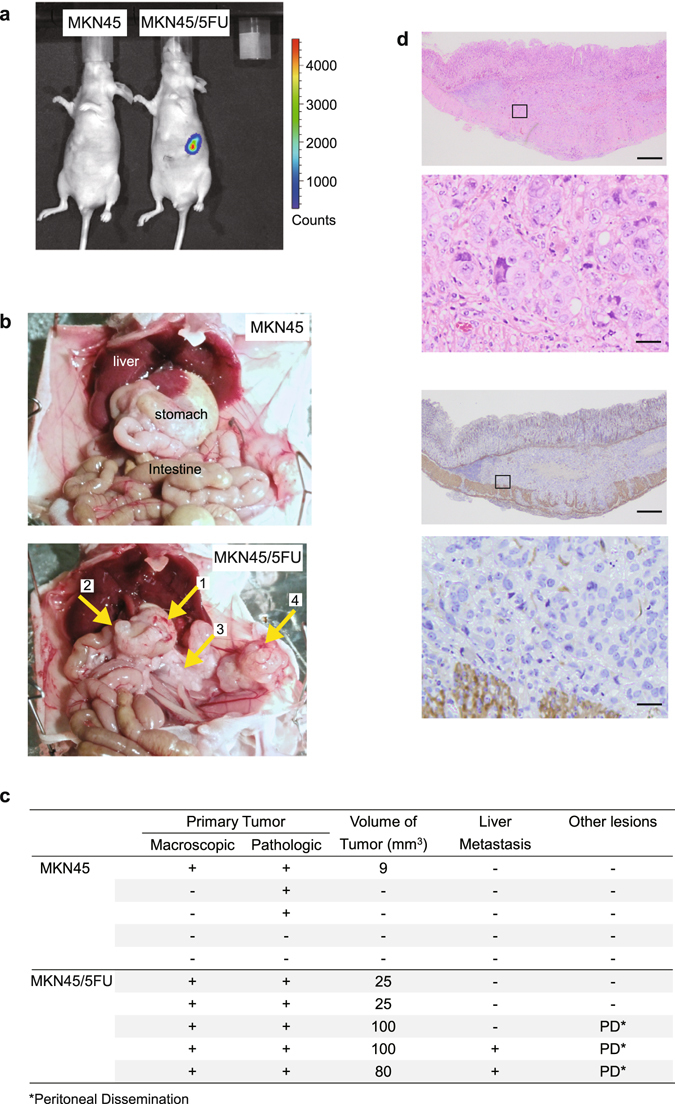
Propagation of MKN45 and MKN45/5FU in OX. (**a**) Detection of OX in the mouse stomach by IVIS. The color bar indicates Charge-Coupled Device (CCD) counts from luciferin. Most CCD counts exceeded noise levels (600) and were far below the CCD saturation (60,000). (**b**) Six weeks after OX of MKN45 cells (top) and MKN45/5FU cells (bottom). Yellow arrows indicate: 1, tumor in the stomach; 2, pyloric regional lymph node metastasis; 3, lymph node metastasis in the visceral peritoneum; and 4, dissemination to the parietal peritoneum. (**c**) Comparison of MKN45 and MKN45/5FU tumorigenicity in OX. Cells (1.0 × 10^6^) were injected into the layer between the submucosa and muscle layers. Six weeks after the cell injection, the mouse organs were pathologically examined. (**d**) Loupe and enlarged views of MKN45/5FU OX between submucosal and proper muscle layers. Top two panels, H&E; and bottom two panels, IHC of α-SMA. Squares in the loupe view indicate the enlarged area shown below. Scale bars for the loupe and enlarged views are 1 mm and 20 μm, respectively.

Although a subcutaneous xenograft of MKN45 and MKN45/5FU cells showed a similar degree of tumorigenicity (Fig. 1d), the CoLA assay suggests that PI3K/AKT/ mechanistic target of rapamycin (mTOR) pathway activation in MKN45/5FU cells may confer a growth advantage (Fig. 2d). To confirm this hypothesis, MKN45 and MKN45/5FU cells were injected by means of “detachment layers by liquid injection”, between the submucosae and proper muscle layers, at which the connectivity of tissues is loose (see Experimental Procedures). Six weeks after transplantation, the MKN45 OX mice showed no detectable tumors, whereas MKN45/5FU OX mice had strikingly large (25–100 mm^3^) tumors in the stomach (Fig. 3a). The OX tumors derived from MKN45/5FU cells in the stomach could metastasize to lymph nodes and the liver, as well as form peritoneal tumors (Fig. 3b and c). H&E staining and immunohistochemistry with α-smooth muscle antigen (α-SMA) showed that the OX tumors in the stomach propagated primarily between the submucosal and muscle layers (Fig. 3d). The “tissue level” and “organ level” OX models differ in that the former precisely mimics the histological location that is associated with the microenvironment. In many previous studies, xenografts were injected close to the serosal layer or into the stomach “wall” without layer-level resolution so as to achieve peritoneal dissemination^20^. Thus, the high frequency of tumor metastases in this study may be due to the high chance that vessels in the submucosal layer were exposed to transplanted tumor cells. With the layer-specific injection method used here, advanced gastric cancer development can be observed more precisely. Together, the aggressive tumorigenicity exhibited by MKN45/5FU cells suggests that selection by 5-FU resulted in the acquisition of a highly malignant potential *in vivo*.

### 5-FU treatment enriches PI3K pathway-activated cell populations in an OX model

**Figure 4:**
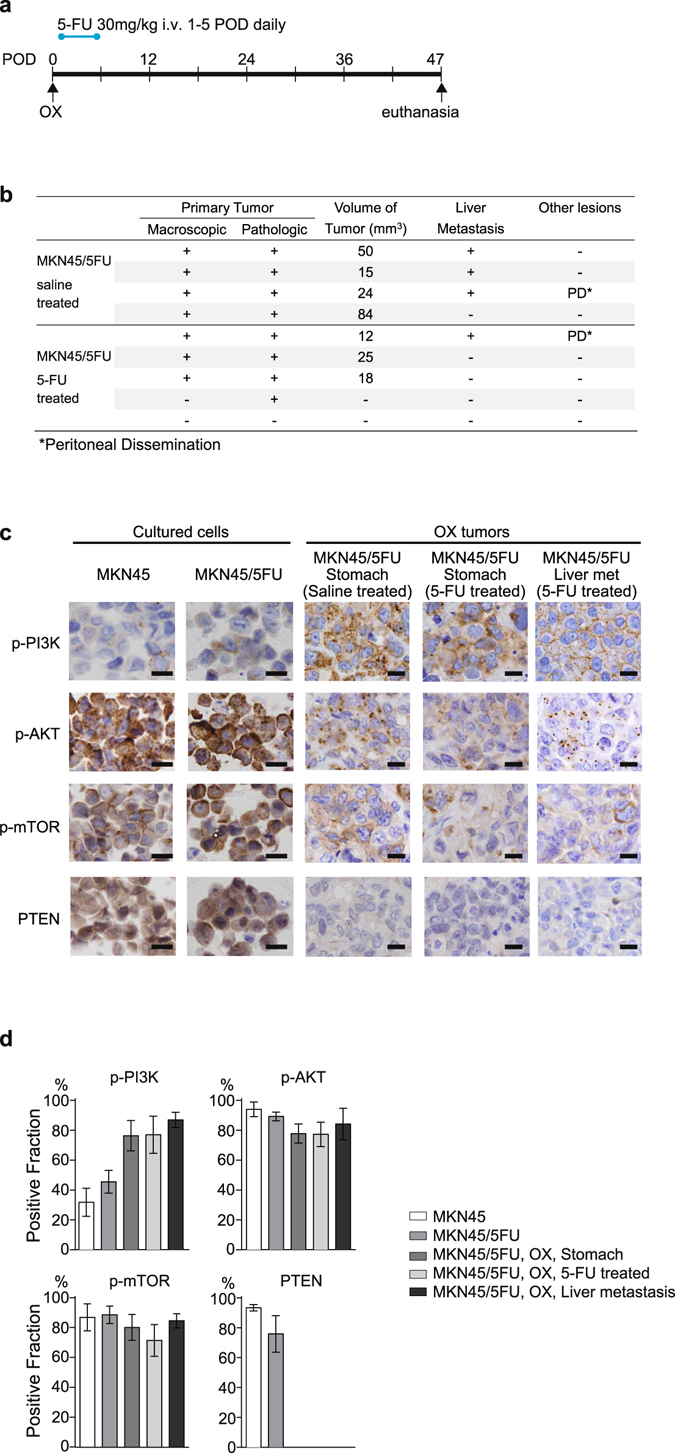
Expression of PI3K pathway proteins *in vivo* and *in vitro*. (**a**) Schematic timeline of 5-FU delivery for MKN45/5FU OX. 5-FU (30 mg/kg/day) was injected daily into the tail vein for 5 days after the initial inoculation. (**b**) Tumor formation by MKN45/5FU OX in the presence and absence of 5-FU. (**c**) Proteins involved in PI3K/Akt/mTOR/PTEN signaling were stained at each stage of MKN45/5FU cell growth. Two columns from the left (MKN45 and MKN45/5FU) are the cultured cells. The right three columns (MKN45/5FU, Stomach; MKN45/5FU, Stomach, 5-FU treated; and MKN45/5FU, Liver met) are OX tumors. MKN45/5FU (Stomach) is the OX tumor grown in the stomach without drug treatment, MKN45/5FU (Stomach, 5-FU treated) is the OX tumor remained after 5-FU treatment, and MKN45/5FU (Liver met) is the metastatic tumor from the OX grown in the stomach (scale bar, 10 μm); (**d**) The positive fraction of each staining is indicated (error bars indicate standard error of the mean for five views).

Next we investigated cancer cell and tumor immunophenotypes according to drug and environmental selection processes. MKN45/5FU OX mice were given a 5-FU infusion daily for 5 days from 1 postoperative day (POD) (Fig. 4a). The 5-FU treatment reduced the size and metastatic potential to some extent (Fig. 4b). To confirm if the “drug-tolerant” population originated from colonies formed in the presence of 5-FU, cultured cells and OX tissues were stained with antibodies against p-PI3K (Fig. 4c). Immunohistochemistry showed that the prevalence of cells that were positive for PI3K pathway components, including p-AKT, p-mTOR, and phosphatase and tensin homolog (PTEN) proteins, changed substantially during the course of tumor development (Fig. 4d). Moreover, the prevalence of p-PI3K -positive cells was significantly increased in MKN45/5FU cells relative to MKN45, and almost all individual MKN45/5FU cells showed a positive staining trend in the subsequent OX and metastatic sites. PTEN expression was almost depleted in OX, indicating that the reduced PTEN levels were further regulated by adaptive microenvironmental factors^21, 22^. Overall, these findings suggest that growth signals via the PI3K/AKT/mTOR pathways could be a therapeutic target for tumor cells that robustly survive after primary 5-FU chemotherapy.

### A *PIK3CA* somatic mutation at codon 707 is unlikely to be the predominant cause of a malignant phenotype acquisition

**Figure 5:**
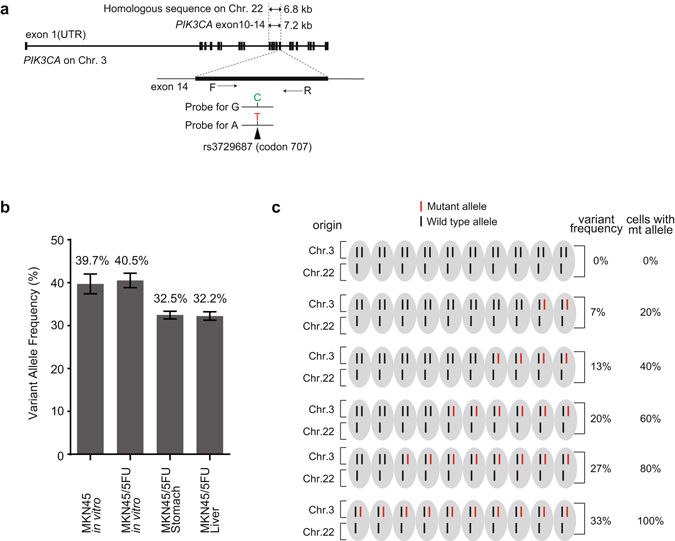
Suppression of OX tumors by 5-FU and PI3K inhibitors. (**a**) Map of *PIK3CA* located on chromosome 3 and a highly homologous pseudogene that spans a 6.8 kb segment on chromosome 22. Codon 707 lies within exon 14, which entirely overlaps the pseudogene sequence such that the mutant allele G > A cannot be distinguished from the pseudogene solely based on dPCR information; (**b**) Prevalence of mutated codon 707 in MKN45/5FU cells under indicated conditions. dPCR was used to reveal the exact number of mutant alleles in each genomic DNA sample. The mean is displayed as a percentage and error bars indicate the standard error of the mean and the range from triplicate experiments. dPCR was performed in triplicate. (**c**) Schematic representation of the cytogenetic status of MKN45 cells and possible variant allele frequency among cells. The mutated *PIK3CA* region is located on chromosome 3 in a region that shares high homology to chromosome 22. Based on the aCGH result showing that the respective region of chromosome 3 was retained while that of chromosome 22 was deleted, the VAF can estimate what fractions of cells may carry the *PIK3CA* mutation.

*PIK3CA* hotspot mutations are thought to increase enzymatic activity to drive a strong oncogenic potential^19^. Indeed, ectopic hotspot mutations can reduce apoptotic activity while enhancing the potential for tumor invasion^23^. Mutations in *PIK3CA* coding exons have been reported in various tumors with broad frequency^19^. The mutation rate of *PIK3CA* in gastric cancer is low relative to other cancer types^19, 24, 25^, and the association between *PIK3CA* mutation and constitutive PI3K activation, or the prognostic impact of these changes, remains controversial^26–28^. The increased sequencing depth and length coverage afforded by NGS allowed the identification of potentially oncogenic non-hotspot mutations^29^. The *PIK3CA* mutation was detected in codon 707 (i.e., E707K), which lies in the catalytic domain encoded on chromosome 3, suggesting a potentially novel oncogenic mutation in gastric cancer^24, 30^. The E707K mutation has been reported in carcinomas arising from the parotid gland^31^ and breast^32^, such that *PIK3CA* mutations can be used to track population enrichment during selection of drug-tolerant cancer cells and cell populations having a malignant phenotype. NGS assessment of variant allele frequency (VAF) for the codon 707 G > A mutation *in vitro* was 31.2 and 30.8% for MKN45 and MKN45/5FU cells, respectively (Supplementary Table 1). To validate these sequencing results and investigate the enrichment of VAFs in cell culture and OX, we designed a pair of primers for digital PCR (dPCR) with a specific probe targeting rs3729687 (Fig. 5a). The mean VAFs of the mutant allele were 39.7, 40.5, 32.5, and 32.2% in MKN45, MKN45/5FU, and MKN45/5FU tumors in the stomach as well as and MKN45/5FU liver metastasis, respectively (Fig. 5b and Supplementary Fig. 2).

The mutated region in *PIK3CA* exon 14 shares a high (>99%) homology with an approximately 7.0 kbp region in chromosome 22 (ref. 27; Fig. 5a). Previous MKN45 karyotyping showed that the long arm of chromosome 3 was retained, whereas one of the chromosome 22 pair was deleted^33^. Our array comparative genomic hybridization (aCGH) also indicated that the copy number of this *PIK3CA* region (3q26.32–3q26.33, including nucleotide 178,932,477–178,939,690, 7,214 bp) was retained in both MKN45 and MKN45/5FU cell types, whereas the copy number for the homologous region on chromosome 22 (nucleotide 17,049,390–17,056,254, 6,865 bp, GEO accession number for the aCGH is GEO: GSE89191; Supplementary Data). These cytogenetic observations helped interpret quantitative measurements of codon 707 mutations by dPCR. Theoretically, the highest VAF for codon 707 on chromosome 3 should be no more than 33.3% if all cells had the mutation (Fig. 5c). Based on this estimate, the dPCR result suggests that cells with the *PIK3CA* codon 707 mutation would dominate during any tumor stage. Since the prevalence of p-PI3K-positive cells was consistent with the degree of malignant phenotype for MKN45 and MKN45/5FU cells, the *PIK3CA* mutation is unlikely to be the predominant cause of the acquisition of tumor malignancy in the case of MKN45/5FU cells. Indeed, we found no codon 707 mutations in another parental/tolerant cell line pair, MKN74 and MKN74/5FU (Supplementary Table 3). Since 5-FU-tolerance is associated with p-PI3K increases, this lack of association between the E707K mutation and p-PI3K status may also support an adaptive mechanism for acquired drug resistance^7, 10^.

### Inhibition of PI3K activation reduces the malignant potential of MKN45/5FU cells

**Figure 6:**
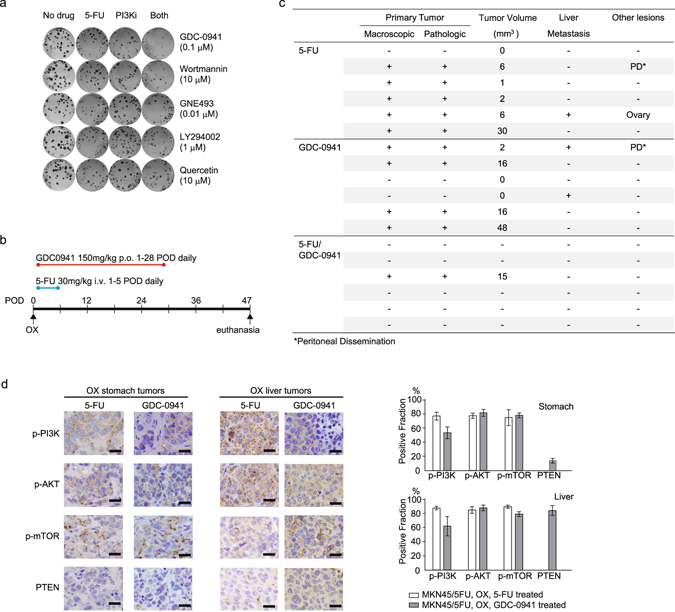
Tumor suppressive effect of 5-FU/GDC-0941 co-administration. (**a**) Colony formation assay for evaluating combined effects of 5-FU and PI3K inhibitors. (**b**) Schematic depiction of the drug administration schedule. GDC-0941 alone, 5-FU alone, or GDC-0941/5-FU were administered beginning on 1 POD. Mice were observed for forty-seven days after OX. (**c**) Tumorigenicity is suppressed by either 5-FU or DGC-0941 to some degree, whereas some metastatic lesions are still observed. Co-administration of 5-FU/GDC-0941 suppressed tumor formation more effectively without visible side effects. Corresponding saline-treated OX showed a high degree of tumor formation that was similar to that shown in Figs 3c and 4b; (**d**) OX tumors treated with GDC-0941 were immunostained with antibodies against PI3K pathway proteins in tissues from the remaining tumor in the stomach and from the liver metastatic site. OX tumor formation following 5-FU/GDC-0941 treatment was not reproducible, so no pathological interpretation was made. The positive fraction of each staining is indicated in the right panel (error bars indicate standard error of the mean for five views). Scale bar, 20 μm.

The finding that genetic alterations had a limited effect on the acquisition of tumor malignancy led us to examine possible inhibitory effects of activated PI3K pathway at protein level. To determine whether PI3K inhibitors can suppress tumors with persistent growth after 5-FU treatment, the PI3K inhibitors LY294002, quercetin, wortmannin, GNE493, and GDC-0941 were tested in a colony formation assay^34–37^. Administration of either 5-FU or PI3K inhibitors alone was associated with modest colony suppression, whereas simultaneous administration of 5-FU and most of the tested PI3K inhibitors had combined effects (Fig. 6a). Since simultaneous administration of 5-FU and GDC-0941 significantly suppressed colony formation, we examined the tumor suppression effect of GDC-0941 in the OX model^38^. GDC-0941 is a very selective, potent class I PI3K inhibitor that targets various forms of p110α, including wild type and mutated versions (E545K and H1047R) that acquired oncogenic activation^39, 40^. Mice with MKN45/5FU OX were treated with intravenous 5-FU followed by GDC-0941 administered orally (Fig. 6b). With no treatment, 100% (9/9) of OX mice exhibited local tumor growth in the stomach, while 5 and 4 of the 9 mice showed liver metastasis and peritoneal dissemination, respectively (Figs 3d and 4b). With 5-FU treatment, 82% (9/11) of OX mice had local tumor growth and 27% (3/11) had metastases (Figs 4b and 6c). Of the mice given the PI3K inhibitor GDC-0941 alone, 67% (4/6) of OX mice had tumor growth, whereas 33% (2/6) and 17% (1/6) of OX mice had liver metastasis and peritoneal dissemination, respectively (Fig. 6c). The combination of 5-FU and GDC-0941 demonstrated striking tumor suppression wherein only 17% (1/6) of OX mice had visible tumors and there was no evidence of metastasis (Fig. 6b and c). Interestingly, the growth suppression assays demonstrated that the 5-FU-tolerant cell lines MKN45/5FU and MKN74/5FU had 3.2- and 2.4-fold higher sensitivity to GDC-0941 than that of respective parental cells (Supplementary Table 3). Moreover, immunohistochemical results for the tumors remaining after treatment showed that GDC-0941 induced different pathway activation from those activated by 5-FU. Here, a significant decrease in p-PI3K-positive cells was apparent, whereas PTEN-positive cells were occasionally found in GDC-0941-treated tumors in the stomach (Fig. 6d). The number of PTEN-positive cells was further increased at liver metastases, where the levels were nearly equal to that of the parental MKN45 cell line (Fig. 4c). Since p-PI3K-positve cells appear to be associated with 5-FU-tolerance, the reduction in the number of the p-PI3K-positive cells seen after GDC-0941 treatment may indicate increased 5-FU effectiveness. Overall, these results suggest that the PI3K inhibitory treatment is more effective when 5-FU treatment enriched cells with PI3K pathway activation.

### GDC-0941 selectively inhibits ribosomal S6 kinase phosphorylation in 5-FU-tolerant cells

**Figure 7:**
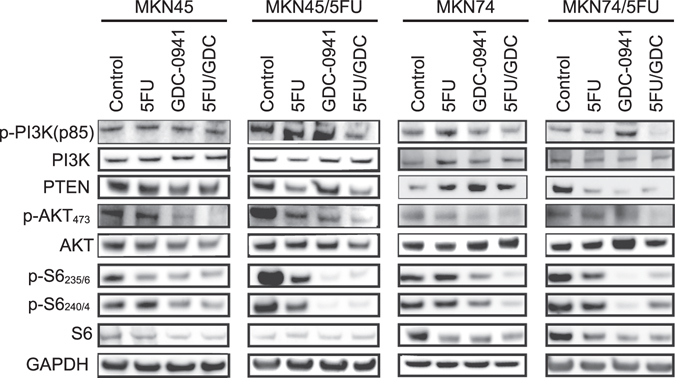
Co-administration of 5-FU and GDC-0941 suppressed S6 kinase phosphorylation in 5-FU-tolerant cell lines. Immunoblot of PI3K pathway proteins in two pairs of 5-FU-tolerant/parental cell lines. Cells were treated with the indicated drugs using GI_50_ concentrations for the respective parental cells. Glyceraldehyde 3-phosphate dehydrogenase (GAPDH) was used as loading controls.

CoLA profiling suggested that 5-FU-tolerant colonies tended to have p-PI3K levels increased with 5-FU concentrations. OX tumor immunohistochemistry supported the hypothesis that p-PI3K-expressing 5-FU-tolerant cells are enriched in the gastric microenvironment. At the bulk cell lysate level in western blots, simultaneous 5-FU/GDC-0941 treatment showed decreased level of p-PI3K in 5-FU-tolerant cells (Fig. 7). The reduction of PTEN levels induced by drug treatment occurred only in 5-FU-tolerant cells; however, the level of PTEN reduction was not as pronounced as what was seen in the OX. Hence, the complete PTEN depletion may be due to the gastric microenvironment. Inhibition of p-AKT was seen in the simultaneous 5-FU/GDC-0941 treatment in parental and tolerant cell lines. Importantly, inhibition of PI3K pathway activation by GDC-0941 was clearly manifested by decreases in ribosomal p70 S6 kinase (S6 kinase) phosphorylation (p-S6 kinase, Ser235/236 and Ser240/244), particularly in the 5-FU-tolerant cells. These results suggest that the S6 kinase, as an mTOR surrogate marker, may also be an important target of GDC-0941 for growth suppression of 5-FU-tolerant subpopulations^41^. However, in contrast to drug-tolerant colonies, cultured cells may still contain a substantial amount of drug-sensitive cells. The western blots showed a modest change in most of the PI3K pathway proteins assessed, except for S6 kinase, which suggests that p-S6 kinase inhibition could occur relatively rapidly in a majority of cultured cells. Indeed, reductions in mTOR were not evident in OX tumors in GDC-0941-treated mice. This outcome could be because western blotting shows actual drug responses at the molecular level by cultured cells, whereas immunohistochemistry of OX tumors represents only tumor cells that remained viable after the therapy.

Although the PI3K pathway appears to be activated in 5-FU-tolerant colonies, Akt and S6 kinase activation are not always concomitantly triggered^41^. However, phosphorylation of S6 kinase is thought to be a major target in PI3K pathway inhibition^38, 42^. As we observed in DTCs, p-PI3K levels in colonies gradually increased with 5-FU treatment. GDC-0941 reduced the 5-FU-tolerant p-PI3K-positive cells by suppressing p-S6 kinase. These results suggest that GDC-0941 treatment would likely be effective against drug-tolerant subpopulations enriched by 5-FU.

In practice, constant activation of p-PI3K in tolerant cells still offers the possibility that PI3K inhibitors could have therapeutic applications regardless of *PIK3CA* mutation status in gastric cancer. GDC-0941 is thought to target p-S6 kinase, which is an mTOR pathway effector *in vitro* and *in vivo* ^38, 42, 43^. Although we found that p-S6 kinase persists with 5-FU administration alone, the addition of GDC-0941 substantially reduced the amount of p-S6 kinase in 5-FU-tolerant/parent pairs even with different *PIK3CA* backgrounds. These results together suggest that 5-FU-based chemotherapy followed by GDC-0941 administration may be a reasonable option for many gastric cancer patients treated with 5-FU, particularly those treated under an adjuvant setting.

## Methods

### Cell lines and drugs

The human gastric cancer cell lines MKN45 and MKN74 were obtained from the RIKEN Cell Bank. Cells were cultured at 37 °C in RPMI 1640 medium supplemented with 10% fetal bovine serum and 5% CO_2_, 80% humidity. Cells were subsequently cultured in the presence of continuous (3 to 5 day) 5-FU exposure followed by increasing concentrations of 5-FU for approximately one year. The timing of the passages and drug concentrations were adjusted based on the growth rate. Here, MKN45 and MKN74 cells were established as the 5-FU-tolerant lines MKN45/5FU and MKN74/5FU, respectively^11^. Except for the colony profiling and western blot described below, we used MKN45/5FU cells for all subsequent studies. Growth suppression and colony formation assays were carried out to confirm the cell line characteristics as previously described^8^. In addition to 5-FU, CIS, DTX, sorafenib, and gefitinib were used in the assays to eliminate cross-resistance.

### Growth inhibition and colony formation assay

For growth inhibition assays, cells were cultured in 96-well microtiter plates at a density of 10,000 cells per well. After incubating for 24 h, drugs were added to each well in a dilution series for 4 h (Supplementary Table 3). WST solution was then added to each well as described previously (Dojindo)^15, 44, 45^. The cell viability represented by 50% growth inhibition concentration (GI_50_) was determined by light absorbance. Colony formation was performed at a density of 100 cells/well of a 6-well plate either in the absence or presence of drugs at 10-fold serial dilutions for 10–14 days. After confirming colony formation in the wells without the drug, colonies were fixed by methanol at −20 °C followed by crystal violet staining. Stained colonies were scanned with a Canon flatbed optical scanner (Canon) at 300 dpi and the resulting image was printed for visual inspection. Colonies with diameters greater than one millimeter were then counted. The 50% colony inhibitory concentration (CoI_50_) was determined with the colony count. All experiments were repeated at least three times independently.

### Colony lysate array

Cells were disseminated in 6-well plates at a density of 100 cells/well under individual drug conditions. Colonies larger than one millimeter in size were picked by hand, washed with PBS, and lysed in a 10 μl of Pink Buffer^46, 47^ in a 200 μL microcentrifuge tube. Cell debris was removed by centrifugation and the supernatant (cell lysate) was transferred to individual wells of a 384-well microtiter plate. The plates were placed in an RPPA microarrayer (Aushon BioSystems) so that each lysate could be transferred onto glass slides embedded in nitrocellulose slides (Grace BioLabs). Since the amount of lysate is much lower, and thus more dilute than lysates for other RPPA applications, we used 5-time printer “pin hits” to capture more protein species in the membrane^8^. Subsequent immunostaining and scanning were performed according to a previously described protocol^8, 17^. The total protein-adjusted protein expression matrix was normalized by mean subtraction for both protein and colony axes^47^. For the concentration-dependent display shown in Fig. 2c, plots were made using an arbitrary unit (a.u.), in which the data deviations were maximized within the vertical axis, resulting in a relative protein expression per protein per drug across drug concentration.

### Orthotopic xenograft

MKN45 and MKN45/5FU cell suspensions were individually prepared at a density of 1.0 × 10^6^ cells/100 μL PBS. Mice were anesthetized with 2% isoflurane gas in a supine position. Then, a 12 mm horizontal incision was made from approximately 5 mm to the left of the median line and 3 mm below the caudal side of the left costal arch. The stomach was gently pulled out, and supported with the left index finger. The cancer cell suspension was then injected into the submucosal layer, which was identified by feeling the release of plunger pressure as the suspension was injected into the rough tissue connective layer (i.e., submucosal layer). After the injection, pressure was applied to the injection site in the gastric wall to ensure that there was no leakage of the injected suspension. Surgical incisions were closed by each layer individually to prevent adhesion after laparotomy.

### PI3K inhibitors

Five compounds known to inhibit PI3K activity, including LY294002 (Sigma-Aldrich)^48, 49^, quercetin (Abcam)^50^, wortmannin (Cell Signaling Technology)^51^, GNE493 (SYNkinase)^34^, and GDC-0941 (Abcam)^38^ were used for initial *in vitro* screening in a colony formation assay. GDC-0941 was selected for tumor suppression assay in an OX model with oral administration.

### Drug treatment for the orthotopic xenograft

Drugs were administered to OX model mice either intravenously (5-FU, Kyowa-Hakko Bio), or orally (GDC-0941, LC Laboratories). A 5-FU solution (200 μl, 25 mg/kg) was injected via the tail vein. GDC-0941 dissolved in 0.5% (w/v) hydroxypropylmethylcellulose (Shin-Etsu Chemical) and 0.2% (v/v) Tween-80 (Sigma-Aldrich) was administered orally at a concentration of 150 mg/kg using a blunt-ended needle inserted via the esophagus. GDC-0941 was delivered once daily for 28 days^52^.

### DNA sequencing of cancer-associated genes

DNA from each tumor was extracted using a Qiagen DNA mini kit (Qiagen). A genomic DNA library was constructed from an Ion AmpliSeq Cancer Panel (49 genes for 190 regions) on Ion PGM (Thermo Fisher Scientific) according to the manufacturer’s protocol. The list of target genes is available at http://www.lifetechnologies.com/order/catalog/product/4471262. Single nucleotide variants were assessed using Torrent Variant Caller (version 4) with a genome viewer and igv software.

### Animal experiments

Six- to eight-week old nude mice (BALB/cAjcl-nu/nu) were obtained from CLEA Japan, Inc. All experiments were performed in accordance with the guideline approval of the Iwate Medical University Ethical Committee for Animal Experiment Regulation (24-029).

### IVIS imaging system

MKN45/5FU cells were transfected with VECTOR-Luc4 and the biological properties were compareble with the parental MKN45 cells. *In vivo* tumor growth was measured by taking images of OX with MKN45/5FU-Luc4 using an IVIS imaging system (Perkin Elmer). Mice were injected with 150 mg/kg of D-luciferin and then anesthetized with 2% isoflurane gas. Fifteen minutes after the D-luciferin injection, the mice were placed on the stage of an IVIS imaging system. The scanning time was five to 10 minutes depending on the experiment, which prevented signal saturation, particularly when multiple lesions were present.

### Immunohistochemistry

Primary antibodies were incubated with the indicated dilution ratio: Phospho-PI3 Kinase p85 (Tyr458)/p55 (Tyr199), 1:100; Phospho-AKT (Ser473), 1:75; Phospho-mTOR (Ser2448) (49F9), 1:75; and PTEN (138G6), 1:450 (Cell Signaling Technology, Danvers, MA). After antigen retrieval (EDTA buffer pH 9 for 30 min at 95 °C), samples were incubated with primary antibody for 60 minutes at room temperature. Peroxidase-labeled anti-rabbit secondary antibody (Histofine® Simple Stain MAX PO, Nichirei Biosciences, Inc.) was then applied for 30 minutes at room temperature. Diaminobenzidine (DAB) was used for colorimetric detection. When anti p-AKT antibody was the primary antibody, samples after antigen retrieval were incubated overnight at 4 °C, followed by a 15 min incubation with peroxidase-labeled anti-rabbit secondary antibody at room temperature. Colorimetric detection was performed using the DAKO’s catalyzed signal amplification (CSA) II Biotin-free Tyramide Signal Amplification System (Agilent Technologies). Samples were deemed to have positive staining when more than 5% of cancer cells were stained.

### Digital PCR

Samples of MKN45 and MKN45/5FU cells, as well as primary OX tumors in the stomach and metastatic OX tumors of the liver that were macroscopically and clearly distinguishable from surrounding mouse tissue (>5 mm) were selected for analysis. Template DNA from scraped cell pellets or WAXFREE (TrimGen)-treated ethanol-fixed paraffin-embedded OX tumor materials was extracted with a QIA DNA Mini kit (Qiagen). OX tumors were dissected manually under a microscope to minimize contamination with mouse tissue. The DNA was subjected to digital PCR reactions in a QuantStudio 3D system (Thermo Fisher Scientific) for which the genome copy number was adjusted to approximately 20,000 for each sample. The specific point mutation was detected with TaqMan MGB probes that were specific for the codon 707 mutation (G > A). All procedures strictly followed the manufacturer’s protocol. Primer and probe sequences for codon 707 were: PIK3CA-codon707F (Forward primer), GGGATGTATTTGAAGCACCTGAAT; PIK3CA-codon707R (Reverse primer), CTGCAGTGAAAAGAGTCTCAAACAC. TaqMan MBG probes (antisense probe): PIK3CA-G-probe (wild type), FAM-ATTGCCTCGACTTGC; and PIK3CA-A-probe (mutant type), VIC-CATTGCCTTGACTTGC.

### Array comparative genomic hybridization

Genomic DNA (1 μg) was extracted from MKN45 and MKN45/5FU cells using a Qiagen Qiamp DNA micro kit. The extracted DNA from MKN45 was labeled using an Agilent Genomic DNA Enzymatic Labeling Kit (Cy3-dUTP), while those DNA from MKN45/5FU was labeled with Cy5-dUTP. The labeled DNA was hybridized to the Agilent Human Genome CGH Microarray Kit 244 K and processed according to the manufacturer’s protocol. Fluorescent array images were acquired with an Agilent DNA microarray scanner (Agilent Technologies). The log2 ratios were corrected for the GC content wave effect using 1 Mb windows for genome GC content. Outliers were smoothed and the normalized log2 ratios were segmented using circular binary segmentation analysis in Partek Genomics Suite v6.4 (Partek Inc.). Genomic segmentation algorithm employed to find segments meeting three criteria: 1) breakpoints (region boundaries) were chosen to give optimal statistical significance (P < 0.001); 2) detected regions must contain a minimum of 10 probes; and 3) segments with relative copy numbers higher than 2.3 or lower than 1.7 to produce CN status such as “Gain” or “Loss”. The entire microarray data set is available at http://www.ncbi.nlm.nih.gov/geo/info/linking.html under the data series accession number GSE89191.

### Statistical analysis

Statistical analyses were performed using either JMP (SAS Institute, Inc.) or GraphPad Prism (GraphPad Software, Inc.) software. For group mean comparison, either Student’s t-test or Mann-Whitney U-test was used. p values less than 0.05 were considered statistically significant.

### Western blotting

Cells were grown to 70% confluence in the presence of 5-FU and GDC-0941, either alone or together, or a PI3K/mTOR inhibitor for 24 hours at 37 °C in an ordinary incubator. Drug concentrations were optimized based on the 48 h GI_50_ value obtained from growth suppression assays (Supplementary Table 3). In the present study, approximately the 48 h GI_75_ value of parental cells for each drug was used for 24 h drug exposure. Proteins were separated according to molecular weight and transferred to a nitrocellulose membrane with a semi-dry format apparatus (iBlot, Invitrogen). The nitrocellulose membrane was then incubated with the specific primary antibodies listed in the Supplementary Table 2 followed by a species-specific secondary antibody conjugated with luciferase. The band was subsequently developed with luciferin substrate according to the manufacturer’s protocol.

## Electronic supplementary material

Supplementary Information

Dataset 1

## References

[1] Cunningham D (2006). Perioperative chemotherapy versus surgery alone for resectable gastroesophageal cancer. N Engl J Med.

[2] Macdonald JS (2001). Chemoradiotherapy after surgery compared with surgery alone for adenocarcinoma of the stomach or gastroesophageal junction. N Engl J Med.

[3] Sakuramoto S (2007). Adjuvant chemotherapy for gastric cancer with S-1, an oral fluoropyrimidine. N Engl J Med.

[4] Brar SS (2014). Processes of care in the multidisciplinary treatment of gastric cancer: results of a RAND/UCLA expert panel. JAMA Surg.

[5] Hata AN (2016). Tumor cells can follow distinct evolutionary paths to become resistant to epidermal growth factor receptor inhibition. Nat Med.

[6] Yu M (2014). Cancer therapy. *Ex vivo* culture of circulating breast tumor cells for individualized testing of drug susceptibility. Science.

[7] Kugel CH, Aplin AE (2014). Adaptive resistance to RAF inhibitors in melanoma. Pigment Cell Melanoma Res.

[8] Kume K (2016). alpha-Amanitin Restrains Cancer Relapse from Drug-Tolerant Cell Subpopulations via TAF15. Sci Rep.

[9] Tomasetti C, Vogelstein B (2015). Cancer etiology. Variation in cancer risk among tissues can be explained by the number of stem cell divisions. Science.

[10] Muranen T (2012). Inhibition of PI3K/mTOR leads to adaptive resistance in matrix-attached cancer cells. Cancer Cell.

[11] Nakamura A (2014). Enhancement of 5-fluorouracil-induced cytotoxicity by leucovorin in 5-fluorouracil-resistant gastric cancer cells with upregulated expression of thymidylate synthase. Gastric Cancer.

[12] Holohan C, Van Schaeybroeck S, Longley DB, Johnston PG (2013). Cancer drug resistance: an evolving paradigm. Nat Rev Cancer.

[13] Valent P (2012). Cancer stem cell definitions and terminology: the devil is in the details. Nat Rev Cancer.

[14] Jones S (2008). Comparative lesion sequencing provides insights into tumor evolution. Proc Natl Acad Sci USA.

[15] Matsuo T (2013). Evaluation of chemosensitivity prediction using quantitative dose-response curve classification for highly advanced/relapsed gastric cancer. World J Surg Oncol.

[16] Kume K (2016). Systematic Protein Level Regulation via Degradation Machinery Induced by Genotoxic Drugs. J Proteome Res.

[17] Spurrier B, Ramalingam S, Nishizuka S (2008). Reverse-phase protein lysate microarrays for cell signaling analysis. Nat Protoc.

[18] Zhu Y (2014). Overexpression of CD133 enhances chemoresistance to 5-fluorouracil by activating the PI3K/Akt/p70S6K pathway in gastric cancer cells. Oncol Rep.

[19] Liu S, Knapp S, Ahmed AA (2014). The structural basis of PI3K cancer mutations: from mechanism to therapy. Cancer Res.

[20] Takei Y, Takigahira M, Mihara K, Tarumi Y, Yanagihara K (2011). The metastasis-associated microRNA miR-516a-3p is a novel therapeutic target for inhibiting peritoneal dissemination of human scirrhous gastric cancer. Cancer Res.

[21] Miething C (2014). PTEN action in leukaemia dictated by the tissue microenvironment. Nature.

[22] Zhang L (2015). Microenvironment-induced PTEN loss by exosomal microRNA primes brain metastasis outgrowth. Nature.

[23] Huang CH (2007). The structure of a human p110alpha/p85alpha complex elucidates the effects of oncogenic PI3Kalpha mutations. Science.

[24] Atlas Research N (2014). Comprehensive molecular characterization of gastric adenocarcinoma. Nature.

[25] Samuels Y, Velculescu VE (2004). Oncogenic mutations of PIK3CA in human cancers. Cell Cycle.

[26] Kalinsky K (2009). PIK3CA mutation associates with improved outcome in breast cancer. Clin Cancer Res.

[27] Ogino S (2009). PIK3CA mutation is associated with poor prognosis among patients with curatively resected colon cancer. J Clin Oncol.

[28] Zardavas D, Phillips WA, Loi S (2014). PIK3CA mutations in breast cancer: reconciling findings from preclinical and clinical data. Breast Cancer Res.

[29] Dogruluk T (2015). Identification of Variant-Specific Functions of PIK3CA by Rapid Phenotyping of Rare Mutations. Cancer Res.

[30] Rudd ML (2011). A unique spectrum of somatic PIK3CA (p110alpha) mutations within primary endometrial carcinomas. Clin Cancer Res.

[31] Grunewald I (2015). Targeted next generation sequencing of parotid gland cancer uncovers genetic heterogeneity. Oncotarget.

[32] Troxell ML (2010). High prevalence of PIK3CA/AKT pathway mutations in papillary neoplasms of the breast. Mod Pathol.

[33] Rege-Cambrin G (1992). Karyotypic analysis of gastric carcinoma cell lines carrying an amplified c-met oncogene. Cancer Genet Cytogenet.

[34] Sutherlin DP (2010). Discovery of (thienopyrimidin-2-yl)aminopyrimidines as potent, selective, and orally available pan-PI3-kinase and dual pan-PI3-kinase/mTOR inhibitors for the treatment of cancer. J Med Chem.

[35] Thorpe LM, Yuzugullu H, Zhao JJ (2015). PI3K in cancer: divergent roles of isoforms, modes of activation and therapeutic targeting. Nat Rev Cancer.

[36] Workman P, Clarke PA, Raynaud FI, van Montfort RL (2010). Drugging the PI3 kinome: from chemical tools to drugs in the clinic. Cancer Res.

[37] Yap TA, Bjerke L, Clarke PA, Workman P (2015). Drugging PI3K in cancer: refining targets and therapeutic strategies. Curr Opin Pharmacol.

[38] Sarker D (2015). First-in-human phase I study of pictilisib (GDC-0941), a potent pan-class I phosphatidylinositol-3-kinase (PI3K) inhibitor, in patients with advanced solid tumors. Clin Cancer Res.

[39] Folkes AJ (2008). The identification of 2-(1H-indazol-4-yl)-6-(4-methanesulfonyl-piperazin-1-ylmethyl)-4-morpholin-4-yl-t hieno[3,2-d]pyrimidine (GDC-0941) as a potent, selective, orally bioavailable inhibitor of class I PI3 kinase for the treatment of cancer. J Med Chem.

[40] Raynaud FI (2009). Biological properties of potent inhibitors of class I phosphatidylinositide 3-kinases: from PI-103 through PI-540, PI-620 to the oral agent GDC-0941. Mol Cancer Ther.

[41] Bhattacharya B (2012). Pharmacologic synergy between dual phosphoinositide-3-kinase and mammalian target of rapamycin inhibition and 5-fluorouracil in PIK3CA mutant gastric cancer cells. Cancer Biol Ther.

[42] Haagensen EJ, Kyle S, Beale GS, Maxwell RJ, Newell DR (2012). The synergistic interaction of MEK and PI3K inhibitors is modulated by mTOR inhibition. Br J Cancer.

[43] Elkabets M (2015). AXL mediates resistance to PI3Kalpha inhibition by activating the EGFR/PKC/mTOR axis in head and neck and esophageal squamous cell carcinomas. Cancer Cell.

[44] Endo F (2014). A compensatory role of NF-kappaB to p53 in response to 5-FU-based chemotherapy for gastric cancer cell lines. PLoS One.

[45] Ishida K (2012). Molecular marker identification for relapse prediction in 5-FU-based adjuvant chemotherapy in gastric and colorectal cancers. PLoS One.

[46] Anderson NL, Esquer-Blasco R, Hofmann JP, Anderson NG (1991). A two-dimensional gel database of rat liver proteins useful in gene regulation and drug effects studies. Electrophoresis.

[47] Nishizuka S (2003). Proteomic profiling of the NCI-60 cancer cell lines using new high-density reverse-phase lysate microarrays. Proc Natl Acad Sci USA.

[48] Gharbi SI (2007). Exploring the specificity of the PI3K family inhibitor LY294002. Biochem J.

[49] Stark AK, Sriskantharajah S, Hessel EM, Okkenhaug K (2015). PI3K inhibitors in inflammation, autoimmunity and cancer. Curr Opin Pharmacol.

[50] Walker EH (2000). Structural determinants of phosphoinositide 3-kinase inhibition by wortmannin, LY294002, quercetin, myricetin, and staurosporine. Mol Cell.

[51] Juss JK (2012). Functional redundancy of class I phosphoinositide 3-kinase (PI3K) isoforms in signaling growth factor-mediated human neutrophil survival. PLoS One.

[52] Wallin JJ (2012). GDC-0941, a novel class I selective PI3K inhibitor, enhances the efficacy of docetaxel in human breast cancer models by increasing cell death *in vitro* and *in vivo*. Clin Cancer Res.

