# Supplementary material for: Inhibition of PI3K suppresses propagation of drug-tolerant cancer cell subpopulations enriched by 5-fluorouracil
Source: Sci Rep. 2017 May 23;7:2262. doi: 10.1038/s41598-017-02548-9 (PMC5442158; doi:10.1038/s41598-017-02548-9)
Supplement: Supplementary file 1 — Supplementary Information [file 41598_2017_2548_MOESM1_ESM.pdf]

Supplementary Information

Inhibition of PI3K suppresses propagation of drug-tolerant cancer cell subpopulations enriched by 5-fluorouracil

Kaoru Ishida[1], Chie Ito[1], Yukimi Ohmori[1], Kohei Kume[1,2], Kei A. Sato[1], Yuka Koizumi[1], Akari Konta[1], Takeshi Iwaya[1], Mamoru Nukatsuka[3], Takashi Kobunai[3], Teiji Takechi[3], and Satoshi S. Nishizuka[1,2,4]

[1]*Molecular Therapeutics Laboratory, Department of Surgery, Iwate Medical University School of Medicine, Morioka, Iwate 020-8505, Japan.*

[2]*Division of Biomedical Research and Development, Institute of Biomedical Science, Iwate Medical University, Morioka, Iwate 020-8505, Japan.*

[3]*Translational Research Laboratory, Taiho Pharmaceutical Co., Ltd., Tokushima, Tokushima 771-0194, Japan.*

[4]*Center for Applied Proteomics and Molecular Medicine, Institute for Advanced Biomedical Research, George Mason University, Manassas, Virginia 20110, United States*

Correspondence and requests for materials should be addressed to S.S.N. (email: snishizu@iwate-med.ac.jp).

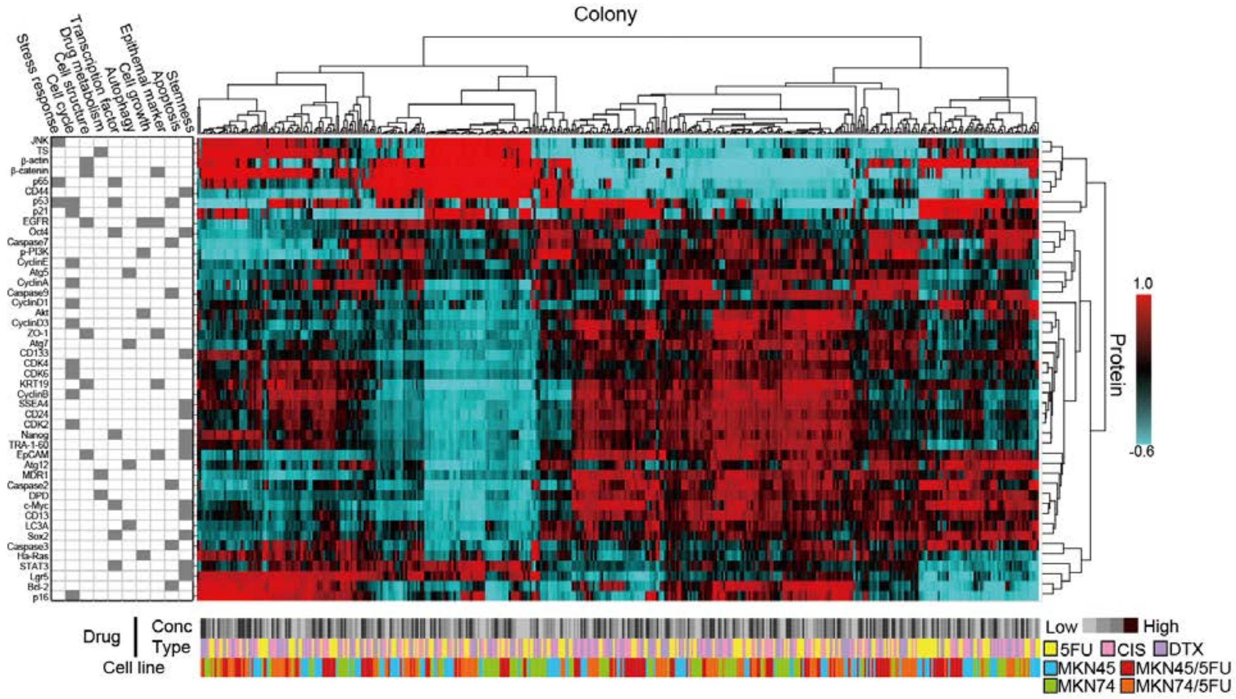

Supplementary Figure 1 | Hierarchical clustering of DTCs. A heatmap was generated based on colonies (horizontal axis) and proteins (vertical axis). Both axes were normalized by mean-subtraction. Distance calculation is based on the correlation coefficient and clusters were constructed with average linkage. Left tiles indicate the representative function of each protein tested in the present study. The three horizontal strips indicate drug concentration (top), drug type (middle), and cell line type. The drug concentration is indicated in grayscale, and the drug type and cell lines are indicated in color. CIS, cisplatin; DTX, docetaxel.

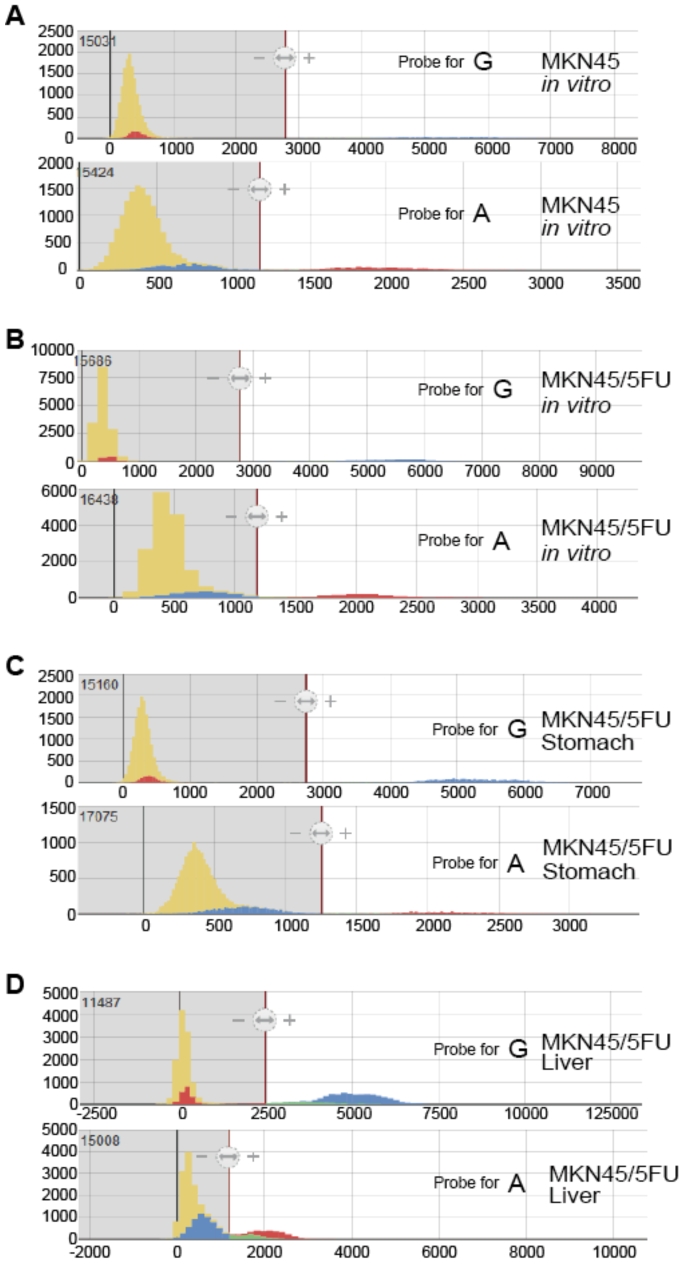

Supplementary Figure 2 | Digital PCR detection of *PIK3CA* codon 707 in MKN45 and MKN45/5FU cells. Each pair of panels represents wild type (probe for G, top) and mutant type (probe for A, bottom). Samples from stomach and liver were derived only from MKN45/5FU cells because the OX tumors from MKN45 showed no significant growth in the stomach.

(A) MKN45 and (B) MKN45/5FU cells in culture; (C) MKN45/5FU transplants in the stomach (primary tumor) and (D) liver (metastatic tumor).

Supplementary Table 1 | Mutations Detected in MKN45 and MKN45/5FU Gastric Cancer Cell Lines

Cell Lines	Chromosome	Symbol	Genotype	Reference	Variant	Var Freq	Total Cov	Var Cov
MKN45								
	3	PIK3CA	G/A	G	A	31.2	397	124
	4	FGFR3	A/A	G	A	98.2	113	111
	4	PDGFRA	G/G	A	G	100	399	399
	4	KDR	A/A	T	A	97.5	398	388
	5	APC	A/A	G	A	95.4	303	289
	10	RET	T/T	G	T	100	389	389
	17	TP53	G/A	G	A	64.4	216	139
MKN45<br>/5FU								
	3	PIK3CA	G/A	G	A	30.8	338	104
	4	FGFR3	A/A	G	A	100	160	160
	4	PDGFRA	G/G	A	G	100	399	399
	4	KDR	A/A	T	A	97	399	387
	5	APC	A/A	G	A	94.5	398	376
	10	RET	T/T	G	T	100	397	397
	17	TP53	G/A	G	A	62.3	239	149

Var Freq, Variant Frequency; Total Cov, Total Coverage; Var Cov, Variant Coverage.

Supplementary Table 2 | List of antibodies

	Protein	Category	Vendor	Catalog no.	Dilution	Application
1	Akt	Cell Growth	CST	9272	1:100	CoLA
					1:1000	WB
2	Atg5	Autophagy	CST	8540	1:100	CoLA
3	Atg7	Autophagy	CST	2631	1:100	CoLA
4	Atg12	Autophagy	CST	4180	1:100	CoLA
5	β-actin	Cell structure	NeoMarkers	MS-1295	1:200	CoLA
6	β-catenin	Epithelial marker<br>Cell structure	CST	9582	1:100	CoLA
7	Bcl-2	Apoptosis	BD	610538	1:100	CoLA
8	Caspase2	Apoptosis	BD	611022	1:100	CoLA
9	Caspase3	Apoptosis	CST	9662	1:100	CoLA
10	Caspase7	Apoptosis	CST	9492	1:100	CoLA
11	Caspase9	Apoptosis	CST	9502	1:100	CoLA
12	CD13	Stemness	Santa Cruz	sc-13536	1:100	CoLA
13	CD133	Stemness	CST	3663	1:100	CoLA
14	CD24	Stemness	Santa Cruz	sc-70598	1:100	CoLA
15	CD44	Stemness	CST	3570	1:100	CoLA
16	CDK2	Cell cycle	BD	610145	1:200	CoLA
17	CDK4	Cell cycle	BD	610147	1:200	CoLA
18	CDK6	Cell cycle	CST	3136	1:200	CoLA
19	Cyclin A	Cell cycle	BD	611268	1:200	CoLA
20	Cyclin B	Cell cycle	BD	610219	1:250	CoLA
21	Cyclin D1	Cell cycle	CST	2926	1:250	CoLA
22	Cyclin D3	Cell cycle	CST	2936	1:250	CoLA
23	Cyclin E	Cell cycle	CST	4129	1:250	CoLA
24	c-MYC	Stemness<br>Transcription factor	Santa Cruz	sc-40	1:100	CoLA
25	dihydropyrimidine dehydrogenase	Drug metabolism	Generated by TAIHO	N/A	1:100	CoLA
26	EGFR	Cell Growth<br>Epithelial marker<br>Cell structure	CST	2232	1:100	CoLA
					1:1000	WB
27	EpCAM	Stemness<br>Cell structure<br>Epithelial marker	Santa Cruz	sc-71057	1:100	CoLA
28	ERK1/2	Cell Growth	BD	612358	1:1000	WB
29	GAPDH	Internal Control	CST	2118	1:1000	WB
30	Ha-Ras	Cell Growth	BD	610001	1:1000	CoLA
31	JNK	Stress response	BD	610627	1:100	CoLA
32	KRT19	Epithelial marker<br>Cell structure	Thermo	MS-377	1:1000	CoLA
33	LC3A	Autophagy	CST	4599	1:100	CoLA
34	LGR5	Stemness	BD	562713	1:100	CoLA
35	MDR1	Drug metabolism	Sigma	P7965	1:100	CoLA
36	NANOG	Stemness	CST	4903	1:100	CoLA

		Transcription factor				
37	Oct-4	Stemness Transcription factor	CST	2840	1:100	CoLA
38	p16	Cell cycle	Santa Cruz	sc-468	1:100	CoLA
39	p21	Cell cycle	CST	2946	1:200	CoLA
40	p53	Stress response Transcription factor Cell cycle Apoptosis	NeoMarkers	MS-187	1:200	CoLA
41	p65	Stress response Transcription factor	CST	3034	1:100	CoLA
42	p-AKT(Ser473)	Cell Growth	CST	9271	1:1000	WB
					1:75	IHC
43	PI3K	Cell Growth	CST	4292	1:1000	WB
44	p-mTOR(Ser2448)	Cell Growth	CST	2971	1:75	IHC
45	p-PI3K(p85)	Cell Growth	CST	4228	1:100	CoLA
					1:1000	WB
					1:100	IHC
46	p-S6(235/6)	Cell Growth	CST	4858	1:1000	WB
47	p-S6(240/4)	Cell Growth	CST	5364	1:1000	WB
48	PTEN	Cell Growth	CST	9559	1:1000	WB
					1:450	IHC
49	pP70S6	Cell Growth	CST	2708	1:1000	WB
50	Sox2	Stemness Transcription factor	CST	3579	1:200	CoLA
51	SSEA4	Stemness	CST	4755	1:100	CoLA
52	STAT3	Stemness Transcription factor	CST	9132	1:100	CoLA
53	TRA-1-60	Stemness	CST	4746	1:100	CoLA
54	Thymidylate Synthase	Drug metabolism	Generated by TAIHO	NA	1:100	CoLA
55	ZO-1	Epithelial	CST	5406	1:100	CoLA

CST, Cell Signaling Technology; WB, Western Blot; CoLA, Colony Lysate Array; NA, Not Applicable.

Supplementary Table 3 | 50% Growth Inhibitory Concentration and Mutational Status of *PIK3CA* in Parent and 5-FU-Tolerant Pairs

Cell Lines	GI_{50}		Codons		
	5-FU	GDC-0941	545	1047	707
MKN45	21.4	4.1	wt	wt	E707K
MKN45/5FU	170.9	1.3	wt	wt	E707K
MKN74	1.4	85	wt	wt	wt
MKN74/5FU	5.5	36	wt	wt	wt

GI_{50} values for 5-FU is μM; and for GDC-0941 is nM.
